# Low Power Adder Based Digital Filter for QRS Detector

**DOI:** 10.1155/2014/405893

**Published:** 2014-05-06

**Authors:** L. Murali, D. Chitra, T. Manigandan

**Affiliations:** ^1^Department of ECE, Hindusthan College of Engineering and Technology, Coimbatore, India; ^2^Department of CSE, P.A. College of Engineering and Technology, Pollachi, India; ^3^P.A. College of Engineering and Technology, Pollachi, India

## Abstract

Most of the Biomedical applications use dedicated processors for the implementation of complex signal processing. Among them, sensor network is also a type, which has the constraint of low power consumption. Since the processing elements are the most copiously used operations in the signal processors, the power consumption of this has the major impact on the system level application. In this paper, we introduce low power concept of transistor stacking to reduce leakage power; and new architectures based on stacking to implement the full adder and its significance at the digital filter level for QRS detector are implemented. The proposed concept has lesser leakage power at the adder as well as filter level with trade-off in other quality metrics of the design. This enabled the design to be dealt with as the low-power corner and can be made adaptable to any level of hierarchical abstractions as per the requirement of the application. The proposed architectures are designed, modeled at RTL level using the Verilog-HDL, and synthesized in Synopsys Design Compiler by mapping the design to 65 nm technology library standard cells.

## 1. Introduction

Signals are natured through the analog and digital types. During the last decade the technology has yielded the powerful, smaller, faster, and cheaper digital computers and special-purposed digital hardware are developed. Such digital circuits are made possible to develop highly sophisticated systems capable of performing complex signal processing functions and tasks which are too expensive and difficult to be handled by the analog circuitry. The digital circuits are cheaper, inexpensive, and reliable in processing the signals, and they also have the ability to provide the hardware with software programmable, which results in flexible system design impacting to achieve higher precisions. Due to the advancement in the digital computer and IC fabrication, the digital signal processing has developed rapidly over the past few decades [1].

The advent of the aging society has made the researchers from both academia and industry show tremendous international interest on the development of the healthcare and monitoring systems. To prevent some of the lifestyle diseases, daily life monitoring is important which makes the raise of number of patients and elderly people requiring the nursing care [2]. As a result new sensing and monitoring devices for healthcare and the use of wearable/wireless devices for clinical applications have been witnessed during the last decade [3]. Such devices are technically called or characterized as body sensor networks. The major challenges in these sensor networks are the lack of integrated signal processing capability and the limited power budget due to the strict design constraint on the device size and weight [4]. Hence there is a need for low power consumption devices for long term in/on body monitoring system.

In this paper, we propose architectures of the processing element for digital filter, which is the extensively used component in complex signal processing applications. The most frequently operated element, computational logic “adder,” is implemented and incorporated in the digital filter of QRS detector for the illustration of impact of the computational component at the application level. The proposed full adder architecture uses the concept of transistor stacking for reducing the leakage power.

The remaining part of the paper is organized as follows.  Section 2 characterizes the low power requirements for the body sensor networks. Architectural logistics for the computational element and digital filter is explained in Section 3. Results and observations are discussed in Section 4. Finally conclusion is provided in Section 5.

## 2. BSN Low Power Requirement

The transmission of health related biomedical data values to the monitoring devices is usually done by the sensors. Sensors are placed either on or inside the body and make use of the intrabody communication techniques for data transmission [5]. The biosensors are categorized into different versions like wearable, wireless, and implantable. The integrated circuits are playing major role in the development of these wearable, wireless, and implantable devices. The ICs have to be developed with rigorous reliability and redundancy for their long term life [3]. The low voltage and low power operations may be obligatory for the battery powered systems which are implantable into the body and will last only for less than the decade number of years. Hence there is a need for low power consumption devices. Because of the major concern of low power requirement regarding the design constraint of these sensor networks, there is a need to develop the architectures that have less operational transitions in the devices. However the processing elements of these sensor processors are the most frequently operated portions and they need to be designed as low powered so that the impact is large in the system level and yield to a longer battery life.

The processing elements of the sensor networks are digital filters, systolic arrays, detectors, and transformers, which in turn contain the computational logical elements. Digital filter of the QRS detector, the most copiously used processing element, is implemented in this paper. Many researchers have proposed the filters, but the suitability to satisfy the design requirement is the challenge. A similar kind of approach was done to achieve the low power in [6], where the processing element of the digital filter, systolic array, was implemented. In this paper we are addressing the issue for the low power filter architecture of the processing block “QRS detector” used in biomedical sensor networks.

## 3. Architecture

**Figure 1:**
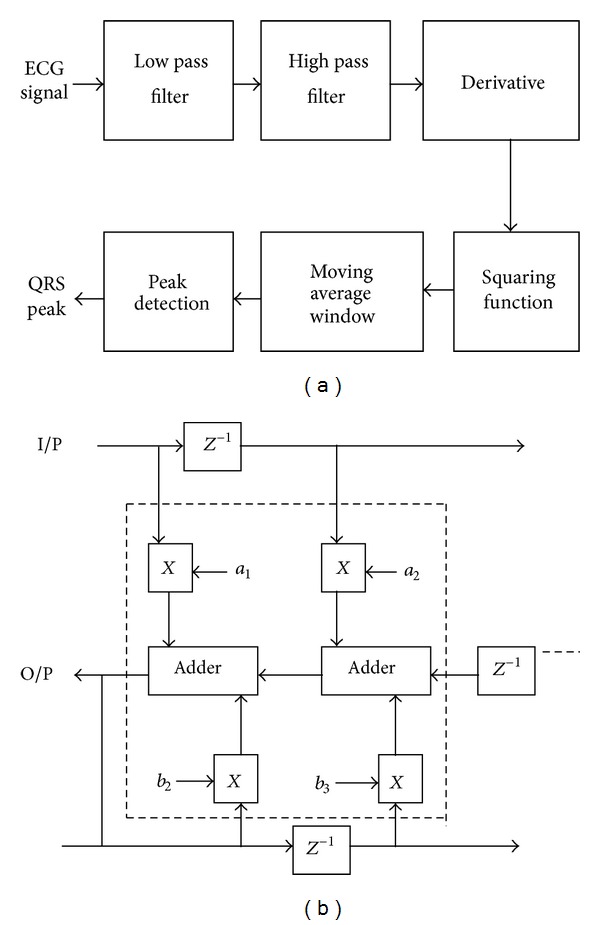
(a) QRS detection steps [6]. (b) Digital filter of QRS detector [6].

To address the impact of computational logic “adder” on the processing element “filter,” the digital filter of the QRS detector is illustrated in this paper for the biomedical signal processing applications. The QRS detector has two stages, namely, preprocessing stage and peak detection stage. The preprocessing stage is composed of various filters for removing the noise and for acquiring the QRS complexes. This information is utilized by the peak detector to arrive at the peaks of QRS complex. Thus the QRS detector has to undergo numerous computations to arrive at the output and a logical hierarchy from adder, systolic array, and filters and then to the peak detectors can be developed. In this paper, the digital filter architecture is implemented.  Figure 1(a) shows the steps involved in detection of QRS complexes and the filter involved in it [6].

The digital filter architecture of Figure 1(b) is modeled using Verilog HDL and synthesized as per the standard ASIC design methodology. This paper addresses the adder architecture and its impact on the digital filter with respect to the design's quality matrices like area, power, and timing.

**Figure 2:**
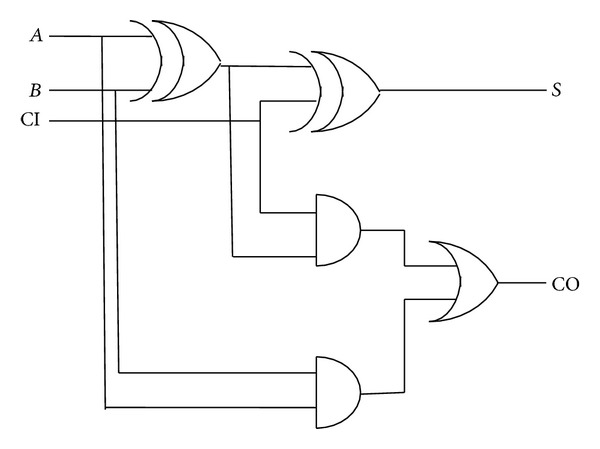
Existing full adder architecture used in existing digital filter implementation.

The digital implementation of the filter is composed of multipliers, adders, and delay elements. Among these, adders are the most copiously used computational logic of the architecture and hence become the prime importance of all. During synthesis of the existing filter architecture of Figure 1(b), Synopsys Design Compiler utilizes the standard adder architecture which is shown in Figure 2, of TSMC technology library, to perform the addition operation.

**Figure 3:**
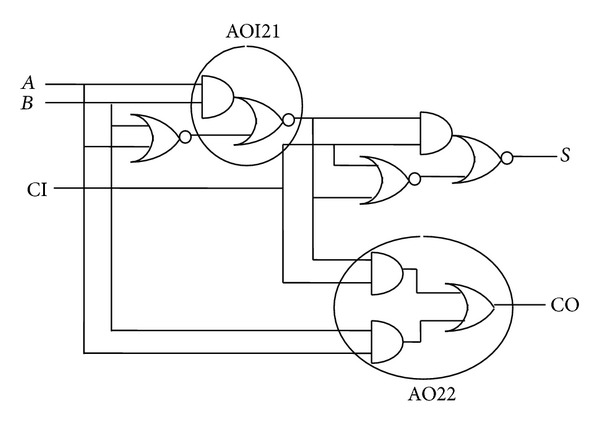
Proposed full adder architecture used in proposed digital filter implementation.

In the proposed filter architecture, the full adder architecture, shown in Figure 3, was built using the concept of transistor stacking and specially designed by considering the low leakage power consumption of the device, as the adders are the most copiously used operators in the design.

**Figure 4:**
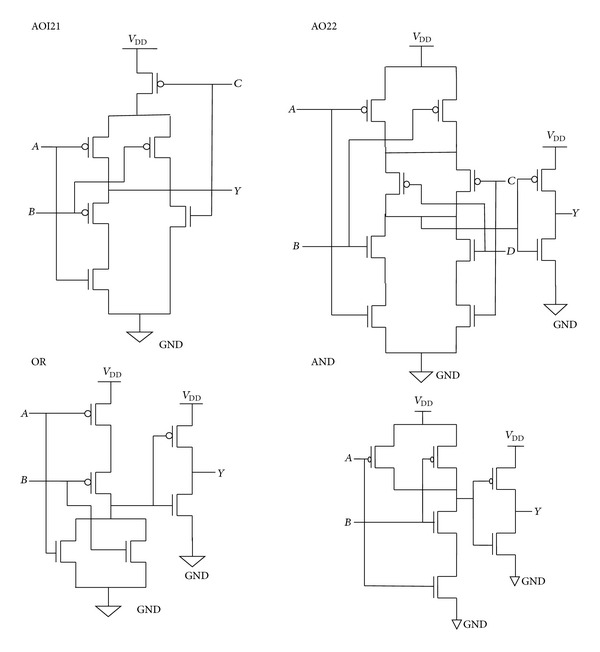
Transistor stack example of logic gates.

Figure 3 shows the proposed full adder architecture. In this adder, the AND-OR-Invert logic cell has more NMOS transistor stacking than the existing full adder architecture cells. The cells of the existing full adder architecture are XOR, AND, and OR logic cells. All these cells have lesser transistor stack than the AOI21 and AO22 logic cells as shown in Figure 4, which are used in the proposed full adder architecture of Figure 3. Expressions for existing and proposed full adders are given in the following equations, respectively:

(1) $$\begin{array}{c} S=A⊕B⊕\text{CI};\;\;\text{CO}=\left(A⊕B\right)\&\;\text{CI}\;|\;\left(A\;\&\;B\right) \end{array}$$

(2) S((A ∣ B−) ∣ (A & B)−)& CI) ∣ (((A ∣ B−) ∣ (A & B)−) ∣ CI)−−CO((A ∣ B−) ∣ (A & B)−)& CI) ∣ (A & CI).

Transistor stacking effect can be analyzed using the subthreshold and gate leakage current. The gate to source voltage (*V*_gs_) is negative when the transistor is closer to the top stack in serially connected transistors stack. And the threshold voltages of top transistors increase due to the reverse biased body to source voltage. Thus the OFF transistors in the stack leak less than the single transistor in the stack [7]. Therefore proposed architecture with higher stacked logic cell increases the ON resistance of transistor stack, thereby reducing the leakage current. The increased ON resistance will reduce the leakage current flow between the supply (*V*_DD_ and *V*_SS_) rails, when the transistors are in standby mode.

From the perspective of design's quality metrics, it can be dealt with as the low-power corner for the ASICs. Such architectures can be utilized as a simple module with plug in and out option in the system level, as they are adaptable to any level of hierarchical abstractions. Such adaptable architectures can be utilized in any application where there is a need to reduce the power consumption. The proposed full adder architecture is compared with conventional 1-bit full adder standard cell of TSMC. The variable bit width architectures are also compared for their behavior at the block level. As mentioned above, the adders are plugged in and out into the digital filter and synthesized. The computational logic “*adder*” impact is observed at the processing element “*filter*” of the processor block “QRS detector.”

## 4. Results and Observations

Both the existing and proposed architectures are designed using Verilog HDL and developed in gate level for synthesis, using the Synopsys Design Compiler (DC) EDA tool. The designs were simulated using the Mentor Graphics ModelSim simulator and verified for functionality with the help of waveform editor. A standard ASIC design methodology was considered to benchmark the results as in [8]. The designs were targeted to the 65 nm technological node and synthesized to get accurate measurement of quality metric's value.

The most frequently operated element, computational logic “adder,” is implemented and incorporated in the digital filter of QRS detector for the biomedical signal processing. The impact of computational logic on the processing element is addressed in the paper. The results of the variable bit width adders are tabulated in Table 1 and their impact in the digital filter can be observed from Table 2.

The tabulated results prove that the proposed architecture has yielded to low leakage power consumption when compared to other architectures by trading off the area. The proposed architecture has enabled new optimization corner (the lowest power with trading for area and performance) for the customer, which will enable opportunities for new markets and applications. From Table 1 it can be observed that the leakage power of the adder has been reduced by 31% in all the varied bit widths of full adder while trading off the area by 21% and proves that the architecture behaves similarly for précised bit widths. Similar to Table 2, at the filter level, the leakage power is reduced by 11% by trading off the area by 5%. Thus the impact ratio of leakage power against the area is increased from 1.5 times in the adder level to 2 times at the system level. This proves that the architecture can be adapted to any level of hierarchical abstractions for low power applications.

## 5. Conclusion

A low power processing element “digital filter” was developed for the biomedical sensor network application. The importance of computational logic “adder” in the complex signal processing applications is proved in this paper, by incorporating the adder in the digital filter of the QRS detector. The adder architecture was built based on the transistor stacking concept to help in cutting off the crossbar current when the device needs to be in standby mode. The architectures were developed with Verilog HDL and synthesized using Design Compiler 2012 by mapping the design into 65 nm technological library node. From the tables it is evident that the proposed design has low leakage power against the counterpart architecture. It also proves, from the increase of impact ratio of leakage power against the area from 1.5 times to 2 times, that the design can be utilized in any level of hierarchical abstraction with low power consumption. Further there is still scope in the reduction of power by applying the concept to other datapath elements.

## Figures and Tables

**Table 1:** Results of variable bit widths adder architecture using TSMC standard full adder cell and proposed full adder cell.

Bit widths	Parameter	Existing	Proposed	% gain
4	Area	40.32	48.96	−21.42
	Timing	0.35	0.57	−62.85
	Dp	9.35	7.47	20.10
	Lp	0.61	0.42	31.05

8	Area	80.63	97.92	−21.44
	Timing	0.62	1.06	−70.96
	Dp	19.38	15.49	20.07
	Lp	1.23	0.84	31.19

16	Area	161.27	195.84	−21.43
	Timing	1.17	2.04	−74.35
	Dp	39.81	31.71	20.34
	Lp	2.46	1.69	31.15

32	Area	322.55	391.68	−21.43
	Timing	2.27	3.99	−75.77
	Dp	80.59	64.18	20.36
	Lp	4.92	3.39	31.14

**Table 2:** Results of existing and proposed digital filter architectures of QRS detector.

Architecture	Existing	Proposed	% gain
Area	3316.68	3490.56	−5.2
Timing	7.35	7.17	2.4
Dp	171.66	164.81	3.98
Lp	36.183	32.200	11.0

Note: area in square microns;

timing in nanoseconds;

Dp = dynamic power in microwatt;

Lp = cell leakage power in microwatt.
